# A Novel Dialkylamino-Functionalized Chalcone, DML6, Inhibits Cervical Cancer Cell Proliferation, In Vitro, via Induction of Oxidative Stress, Intrinsic Apoptosis and Mitotic Catastrophe

**DOI:** 10.3390/molecules26144214

**Published:** 2021-07-11

**Authors:** Jenna M. Len, Noor Hussein, Saloni Malla, Kyle Mcintosh, Rahul Patidar, Manivannan Elangovan, Karthikeyan Chandrabose, N. S. Hari Narayana Moorthy, Manoj Pandey, Dayanidhi Raman, Piyush Trivedi, Amit K. Tiwari

**Affiliations:** 1 Department of Pharmacology and Experimental Therapeutics, College of Pharmacy & Pharmaceutical Sciences, University of Toledo, Toledo, OH 43614, USA; jenna.len@rockets.utoledo.edu (J.M.L.); noor.hussein@rockets.utoledo.edu (N.H.); saloni.malla@rockets.utoledo.edu (S.M.); kyle.mcintosh@rockets.utoledo.edu (K.M.); 2 School of Pharmacy, Devi Ahilya Vishwavidyalaya, Indore 452001, India; r.patidar16@yahoo.in; 3 Department of Pharmacy, Indira Gandhi National Tribal University, Amarkantak 484887, India; karthinobel@gmail.com (K.C.); nshnarayanamoorthy@gmail.com (N.S.H.N.M.); 4 Department of Biomedical Sciences, Cooper Medical School of Rowan University, Camden, NJ 08103, USA; pandey@rowan.edu; 5 Department of Cancer Biology, College of Medicine and Life Sciences, University of Toledo, Toledo, OH 43614, USA; dayanidhi.raman@utoledo.edu; 6 Center of Innovation and Translational Research, Poona College of Pharmacy, Bhartiya Vidyapeeth, Pune 411038, India; piyushtrivedi304@gmail.com

**Keywords:** cervical cancer, chalcone, dialkylamino, intrinsic apoptosis, mitotic catastrophe, drug discovery

## Abstract

In this study, we designed, synthesized and evaluated, in vitro, novel chalcone analogs containing dialkylamino pharmacophores in the cervical cancer cell line, OV2008. The compound, **DML6** was selective and significantly decreased the proliferation of OV2008 and HeLa cells in sub-micromolar concentrations, compared to prostate, lung, colon, breast or human embryonic kidney cell line (HEK293). **DML6**, at 5 μM, arrested the OV2008 cells in the G2 phase. Furthermore, **DML6**, at 5 μM, increased the levels of reactive oxygen species and induced a collapse in the mitochondrial membrane potential, compared to OV2008 cells incubated with a vehicle. **DML6**, at 5 μM, induced intrinsic apoptosis by significantly (1) increasing the levels of the pro-apoptotic proteins, Bak and Bax, and (2) decreasing the levels of l the anti-apoptotic protein, Bcl-2, compared to cell incubated with a vehicle. Furthermore, **DML6**, at 5 and 20 μM, induced the cleavage of caspase-9, followed by subsequent cleavage of the executioner caspases, caspase-3 and caspase-7, which produced OV2008 cell death. Overall, our data suggest that **DML6** is an apoptosis-inducing compound that should undergo further evaluation as a potential treatment for cervical cancer.

## 1. Introduction

Cervical cancer is the fourth-leading cause of cancer-related deaths in women worldwide resulting in an estimated 600,000 new cervical cancer cases and 342,000 deaths, yearly [1]. About 85% of all cervical cancer cases are from underdeveloped or developing nations with 18-fold higher mortality rate compared to developed or high-income countries [2,3]. The majority of cervical cancer is caused primarily due to human papillomavirus (HPV) [4,5]. Other causes of cervical cancer includes infections, smoking, higher number of childbirths and prolonged use of oral contraceptive devices [6,7]. Among all high-risk types, HPV-16 has the highest carcinogenic capacity accounting for 60% of all cervical cancer cases [8,9]. The standard treatment of cervical cancer ranges from cervical conization, hysterectomy and radiotherapy to chemotherapy, depending on the stage of cervical cancer [10]. However, adoption of adjuvant chemotherapy along with chemoradiotherapy to prevent recurrence of locally advanced and metastatic cervical cancers are associated with increased adverse events, morbidity rate and therapeutic failure [11]. Women with locally advanced cervical carcinoma have worse prognosis, poor survival and higher recurrence rate than patients with early-staged cervical cancer [12,13]. Different chemotherapeutic drugs and their combinations have been used to improve the clinical response (CR) and overall survival (OS) in patients with advanced cervical cancer [14]. Despite initial therapeutic response, the majority of patients either undergo relapse or succumb to the disease as a result of resistance to chemotherapy [15]. Chemoresistance is the most important factor that decreases or abrogates the efficacy of chemotherapy in many cancers including advanced cervical cancer, producing an increase in tumor progression, which results in high rates of cancer-related deaths [16,17]. Many factors contribute to chemoresistance, including but not limited to, alterations in DNA damage and repair capacity, and overexpression of ATP-binding cassette (ABC) transporters, notably the ABCB1 transporter [18,19]. Consequently, there is an urgent need for the discovery and development of novel chemotherapeutic drugs that can overcome the aforementioned limitations of therapies used to treat cervical cancer.

**Figure 1:**
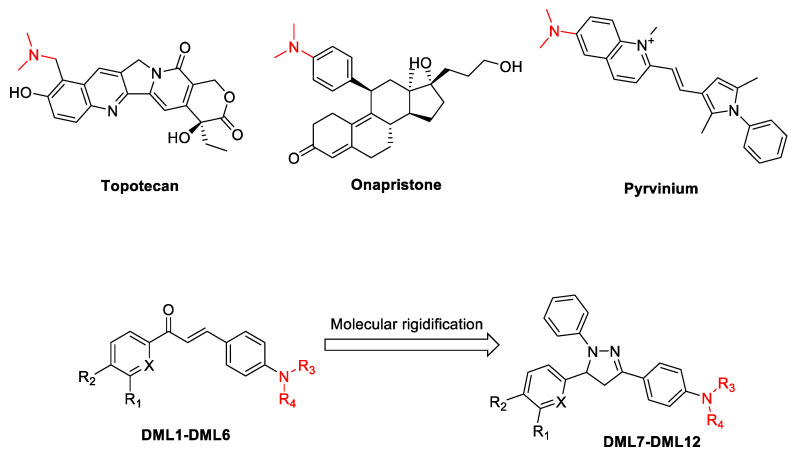
Representative structures of chalcone, dihydropyrazoles and dimethylamine functionalized-derived anticancer compounds.

Chalcones are an essential structural motif that has been extensively used for many years to synthesize novel anticancer drugs [20,21,22,23]. The basic structure of chalcone, which consists of two phenyl rings attached to an α,β-unsaturated carbonyl skeleton at the 1,3 position, provides numerous opportunities for structural alterations to yield novel molecules that selectivity decreases the proliferation of cancer cells [21,23]. Currently, many chalcone derivatives have been synthesized and shown to be efficacious in vitro and in vivo in various cancer cells [20,23]. It has been reported that increasing the rigidity of chalcones produces heterocyclic analogues that have anti-cancer efficacy [24,25]. The 4,5-dihydro-1*H*-pyrazole derivatives synthesized from chalcones have been shown to have potential anticancer efficacy [26]. The addition of a dimethylamino group to chalcone derivatives could be important, as this would improve their solubility and anticancer efficacy [27,28]. Indeed, the dimethylamino function is a structural feature in certain anticancer drugs, such as topotecan [29], pyrvinium [30], and onapristone [31] (Figure 1). Therefore, in this study, we report the synthesis of novel chalcones and their 4,5-dihydro-1*H*-pyrazoles analogues containing the dialkylamine and their in vitro efficacy in various cancer cell lines. Furthermore, we conducted experiments to determine their mechanism of action.

## 2. Results and Discussion

### 2.1. Chemistry

**Scheme 1:**
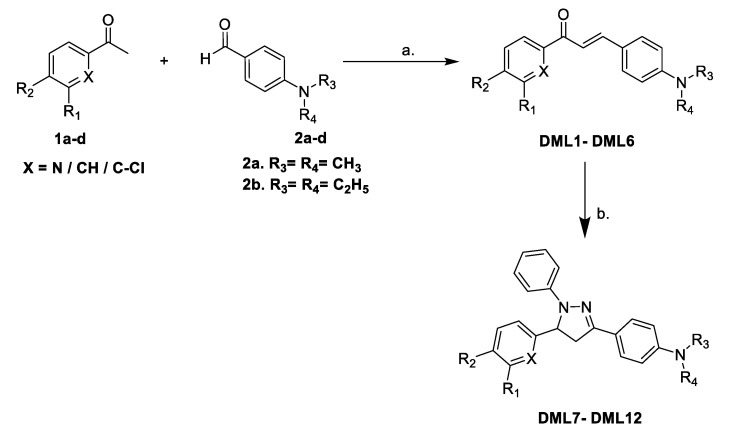
Reagents and conditions: (**a**) 40% aqueous NaOH, EtOH, RT, stirring; (**b**) C_6_H_5_NHNH_2_, CH_3_COOH, reflux, 6 h.

Initially, the target compounds, **DML1**–**DML6**, were synthesized using a simple Claisen-Schmidt condensation reaction, with various substituted acetophenones and 4-dimethylamino- or 4-diethylamino-substituted benzaldehydes in the presence of 40% NaOH solution in EtOH, as described in Scheme 1. Further reaction of the dialkylamino chalcones **DML1**–**DML6** with phenylhydrazine in glacial acetic acid under reflux produced the respective 1,3,5-triphenyl-4,5-dihydro-1*H*-pyrazole **DML7**–**DML12** in good (74 to 86%) yields. The structures of the newly synthesized dialkylamino chalcones **DML1**–**DML6** and their heterocyclic derivatives, 1,3,5-triphenyl-4,5-dihydro-1*H*-pyrazoles **DML7**–**DML12**, were confirmed by microanalyses, FTIR, ^1^H-NMR and mass spectral analysis. All of the synthesized compounds produced satisfactory analytical and spectroscopic, data, which were in full agreement with their proposed structures. The structures, properties and reaction yield of DML compounds are provided in Table 1.

### 2.2. Structure–Activity Relationships for the Dialkylamine Substituted Chalcones and Their Corresponding Dihydropyrazoles, Based on Data Obtained Using the 3-(4,5-Dimethylthiazol-2-yl)-2,5-diphenyltetrazolium Bromide (MTT) Cytotoxicity Assay

All of the synthesized compounds (**DML1** to **DML12**) were evaluated to determine their in vitro antiproliferative efficacy in cervical (OV2008), breast (MDA-MB-231), lung (A549), colon (LOVO), prostate (DU145) and the normal cell lines, human embryonic kidney cells (HEK293), human colon fibroblast cells (CRL1459) and Chinese hamster ovary cells (CHO), using the MTT assay. The newly synthesized compounds were tested at concentrations from 0.1 to 100 μM. The concentration of the tested compounds that produced a 50% inhibition of cell growth (IC_50_) was determined. The calculated IC_50_ values of compounds tested in cancer cell lines are shown in Table 2.

Among the 12 compounds evaluated for antiproliferative efficacy, only three chalcone derivatives, **DML4**, **DML5** and **DML6**, had moderate to good anti-proliferative efficacy in a wide range of cancer cell lines, compared to normal cells. Compound **DML6**, with a chloro-substitution at X and R_2_ positions and a 4-dimethyl amine group, i.e., R_3_ = R_4_ = CH_3_ in chalcone core structure, had significant in vitro antiproliferative efficacy in all of the cancer cell lines, with IC_50_ values ranging from 7.8 to 91.9 μM (Table 2). The most efficacious compound, **DML6**, had an IC_50_ value of 7.8 ± 0, in the OV2008 cervical cancer cells. Similarly, compounds **DML4** and **DML5** were efficacious in the OV2008 cancer cells, with IC_50_ values of 13.8 ± 7 μM and 79.3 ± 29.2 μM, respectively. **DML4**, which had a smaller alkyl substitution at the R_3_ and R_4_ positions, had greater antiproliferative efficacy in the OV2008 cancer cells compared to **DML5**, which had larger alkyl groups at the same position. Based on the cytotoxicity results of DML-4 and DML-5, we found that changing the alkyl chain length of the dialkylamine substituents at the R_3_ and R_4_ positions from methyl to ethyl decreased the antiproliferative efficacy. For the chalcone derivatives, **DML1**–**DML6**, the chloro and methoxy substituents produced the highest efficacy in the R_1_ and R_2_ positions, whereas the 2-pyridyl substituent (X = N) in the chalcone structures decreased the antiproliferative efficacy in OV2008 cells. Similarly, the result of cytotoxicity assay performed in another cervical HeLa cells were similar to that of OV2008 cells (Table S1). **DML6** was the most potent agent with an IC_50_ value of 9.08 ± 0.69 μM, whereas the IC_50_ values of **DML1**, and **DML5** in HeLa cell line were greater than 70 μM, and **DML5** was around 40 μM, suggesting that **DML6** were most effective in cervical cancer.

Among the compounds evaluated in DU145 prostate cancer cells, **DML4** had the highest efficacy, with an IC_50_ value of 22.5 ± 4.9 μM. However, **DML6**, which was the most efficacious compound in OV2008 and HeLa cells, had a low antiproliferative efficacy in DU145 prostate cancer cells, with an IC_50_ value of 96.9 ± 4.4 μM. **DML4**, which has two methoxy groups at the R_1_ and R_2_ position, had the highest efficacy in all five cancer cell lines, whereas **DML5**, with only one methoxy group at the R_2_ positions, had a moderate inhibitory efficacy in the cervical and colon cancer cells. Furthermore, **DML4** significantly decreased the proliferation in breast (MDA-MB-231), lung (A549) and colon (LOVO) cancer cells, with IC_50_ values of 26.7 ± 23.9, 45 ± 22.6, and 66.3 ± 4.6 μM, respectively. None of the dialkylamine functionalized dihydropyrazole derivatives, **DML7**–**DML12**, had efficacy in the cancer cells, even at a maximum concentration of 100 μM. Finally, it is important to note that two of the dimethylamino functionalized chalcone derivatives, **DML4** and **DML6**, inhibited the proliferation of HEK-293 cells at a concentration > 65 μM.

These above results indicate that the chalcone skeleton in compounds **DML1**–**DML6** played an important role in inhibiting the proliferation of the cancer cell lines used in this study. There was only one structural difference between compounds **DML1**–**DML6** and compounds **DML7**–**DML12**: the former six compounds had the chalcone skeleton. Based on the structure-activity relationship of these compounds, the α,β-unsaturated carbonyl system is a key structural characteristic present in the chalcone scaffold that modulates the antiproliferative efficacy. Furthermore, our study indicates that the dialkylamino substitution on the chalcone scaffold increased the antiproliferative efficacy. However, for the dihydropyrazole motif, the same substitution did not significantly increase efficacy.

### 2.3. ***DML6*** Antiproliferative Efficacy and Selectivity on Cervical Cancer Cell Lines

**Figure 2:**
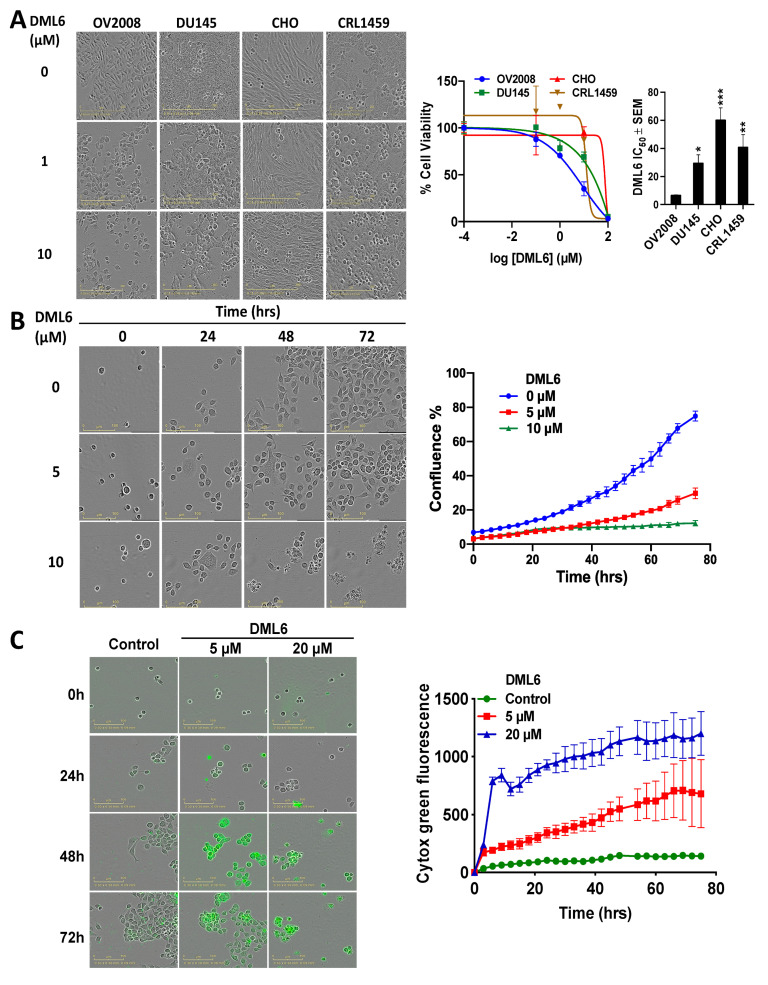
The efficacy and selectivity of DML-6 in OV2008 and DU145 cancer cells compared to the normal cell lines, CHO and CRL-1459. (**A**) The changes in cell morphology produced by the vehicle (0 μM), 5 or 10 μM of **DML6** after 72 h of incubation, the cell-viability curves and the IC_50_ values of **DML6** for these cell lines compared to the normal cell lines. Cell survival was determined using the MTT assay. The IC_50_ values are represented as the means ± SD of three independent experiments performed in triplicate. * *p* < 0.05, ** *p* < 0.01, *** *p* < 0.001. (**B**) The changes in OV2008 cell morphology produced by vehicle (0 μM), 5 or 10 μM of **DML6** after 0, 24, 48 and 72 h of incubation and its confluence graph. (**C**) The real time IncuCyte cytotox green fluorescent reagent assay showing the number of dead OV2008 cancer cells present over time after incubation with vehicle (0 μM), 5 or 10 μM of **DML6**. The data are presented as images showing the fluorescence level at the 0 and 72 h time points. A representative time curve quantitatively summarizing the results at each time point. The data are presented as the means ± SEM of three independent studies. All images were captured in each cell lines after incubation with the vehicle or **DML6** by the IncuCyte^®^ S3 Live-Cell Analysis System at 20× magnification and a representative picture, along with its graph, are shown.

Overall, our MTT results indicate that compound **DML6** was the most optimal candidate for further mechanistic investigations, based on its efficacy in cervical cancer cell lines. In terms of selectivity, **DML6** was significantly less efficacious in decreasing the viability of Chinese hamster ovary cells (CHO) and normal CRL-1459 cells, compared to the cancer cell lines, as illustrated in Figure 2A. Thus, **DML6** significantly inhibited the growth of the cervical and prostate cancer cells. In contrast, **DML6** did not produce significant cytotoxicity in the normal cells, CRL1459 and CHO cells. Figure 2B shows representative pictures of the confluence of OV2008 following incubation with the vehicle, 5 or 20 μM of **DML6**, at 0, 24, 48, and 72 h. The control cells that were incubated with the vehicle grew over time, reaching their highest confluence after 72 hours of incubation (≈70%, Figure 2B). However, the cells incubated with **DML6** grew significantly slower and only had a very low confluence (≈40% and ≈10%, respectively, at 5 and 20 µM of **DML6**, Figure 2B) at 72 hours. A detailed graph illustrating the results for **DML6** was evaluated at each time point to determine the cytotoxicity over time. Similarly, the results of the IncuCyte Cytotox green assay, as seen in Figure 2C indicates that the fluorescence signal emitted by the dead or non-viable cells incubated with vehicle was very low, suggesting that the cells were viable. However, the fluorescent signal increased significantly in OV2008 cells incubated with **DML6**, indicating that **DML6** increased the number of dead cells, i.e., it produced cytotoxicity over time, compared to cells incubated with a vehicle.

### 2.4. ***DML6*** Induces Oxidative Stress in OV2008 Cells

**Figure 3:**
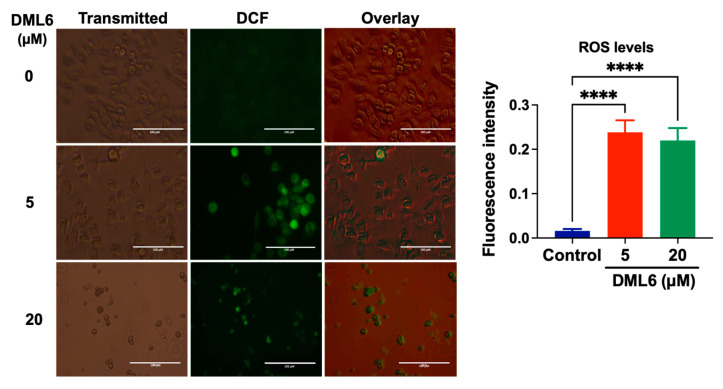
The detection of **DML6**-induced cellular oxidative stress by DCF in OV2008 cells. Representative images of fluorescent DCFDA levels following incubation with the vehicle (control) (0 μM), 5 or 20 μM of **DML6** for 24 h. The images were taken using an EVOS digital fluorescent microscope at 40× magnification. A histogram quantitatively summarizing the change in the % of fluorescence intensity of DCFDA in cells incubated with 5 and 20 μM of **DML6** as compared to cells incubated with the vehicle, is also shown. **** *p* < 0.0001. The experiment was repeated in triplicate for each cell line. Scale bar: 100 μM.

The total intracellular level of reactive oxygen species (ROS) was determined by staining the cells with 2,7-dichlorofluorescein diacetate (H2DCFDA). In this assay, cellular esterases cleave the nonfluorescent H2DCFDA molecule to yield H2DCF, by removing the lipophilic moiety (a diacetate group) [20]. Subsequently, H2DCF is oxidized by ROS to 2′-7′dichlorofluorescein (DCF), which is a highly fluorescent dye [32]. ROS levels were quantified based on the fluorescence level of DCF detected using EVOS microscope. The levels of DCF fluorescence were significantly higher in cells incubated with 5 or 20 μM **DML6**, compared to cells incubated with the vehicle (Figure 3), indicating that **DML6** induced the formation of ROS.

### 2.5. ***DML6*** Arrests the Cell Cycle of OV2008 at G2 Phase

**Figure 4:**
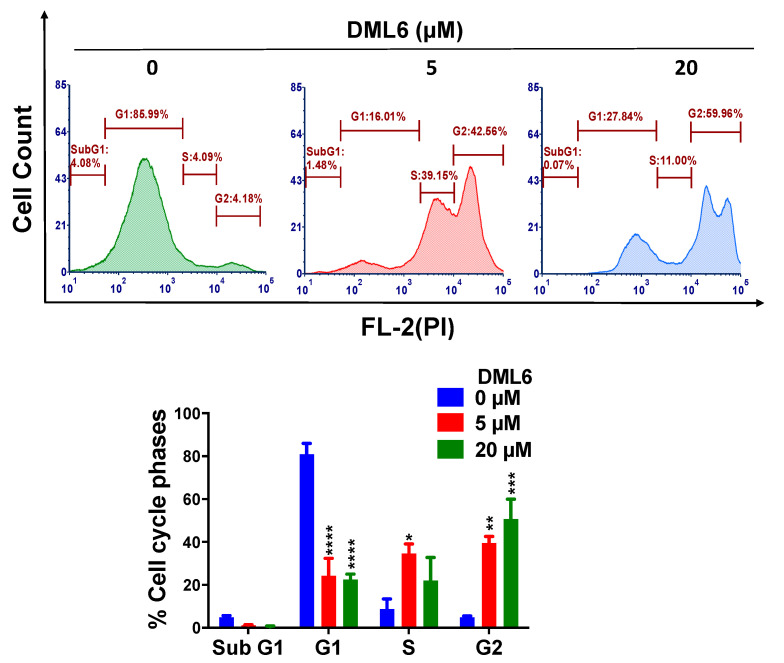
The effect of **DML6** on the cell cycle. A representative figure illustrating the effect of **DML6** on the distribution of the OV2008 cell populations in the cell cycle phases. OV2008 cells were incubated with the vehicle, 5 or 20 μM of **DML6** for 24 h and were subjected to cell cycle analysis using flow cytometry with propidium iodide (PI) (X-axis)/cell counts(Y-axis); A histogram quantitatively summarizing the change in the % of cells in each phase of the cell cycle due to **DML6**, is also shown. * *p* < 0.05, ** *p* < 0.01, *** *p* < 0.001, **** *p* < 0.0001. The data represent the means ± SEM of three independent experiments performed in triplicate.

To further determine the mechanisms by which **DML6** inhibits cervical cancer cell proliferation, a cell cycle analysis was conducted using flow cytometry cell cycle analysis with propidium iodide (PI). **DML6** produced a significant concentration-dependent increase in the percentage of cells in the G2 phase in OV2008 cells. The percentage of cells in the G2 phase increased from 4.18% in cells incubated with the vehicle to 42.56 % and 59.96% with 5 or 20 μM of **DML6**, respectively (*p* < 0.01, *p* < 0.001, respectively; Figure 4). Furthermore, **DML6** significantly (*p* < 0.0001) decreased the percentage of cells in the G1 phase from 85.99% in the cells incubated with the vehicle, to 16.01% and 27.84% after incubation with 5 or 20 μM, respectively, of **DML6** (Figure 4). **DML6**, at 5 µM, significantly (* *p* < 0.05) increased the percentage of cells in the S phase. However, 20 µM of **DML6** did not significantly increase the percentage of cells in the S phase. Finally, neither 5 or 10 µM of **DML6** significantly altered the percentage of cells in the sub G1 phase.

### 2.6. ***DML6*** Induces Mitotic Catastrophe and Apoptosis in OV2008 Cells

**Figure 5:**
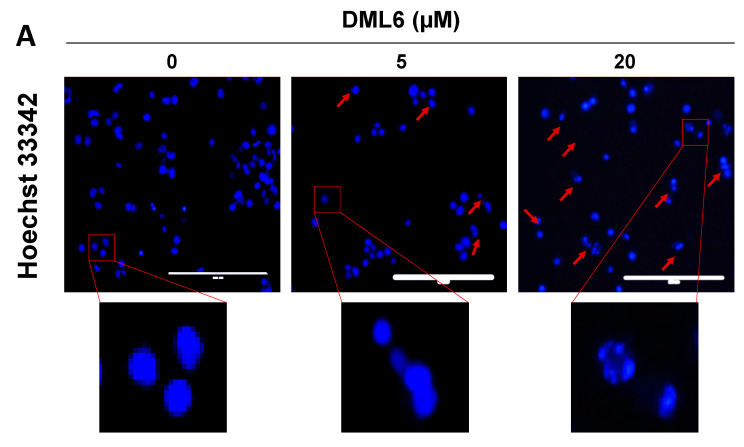

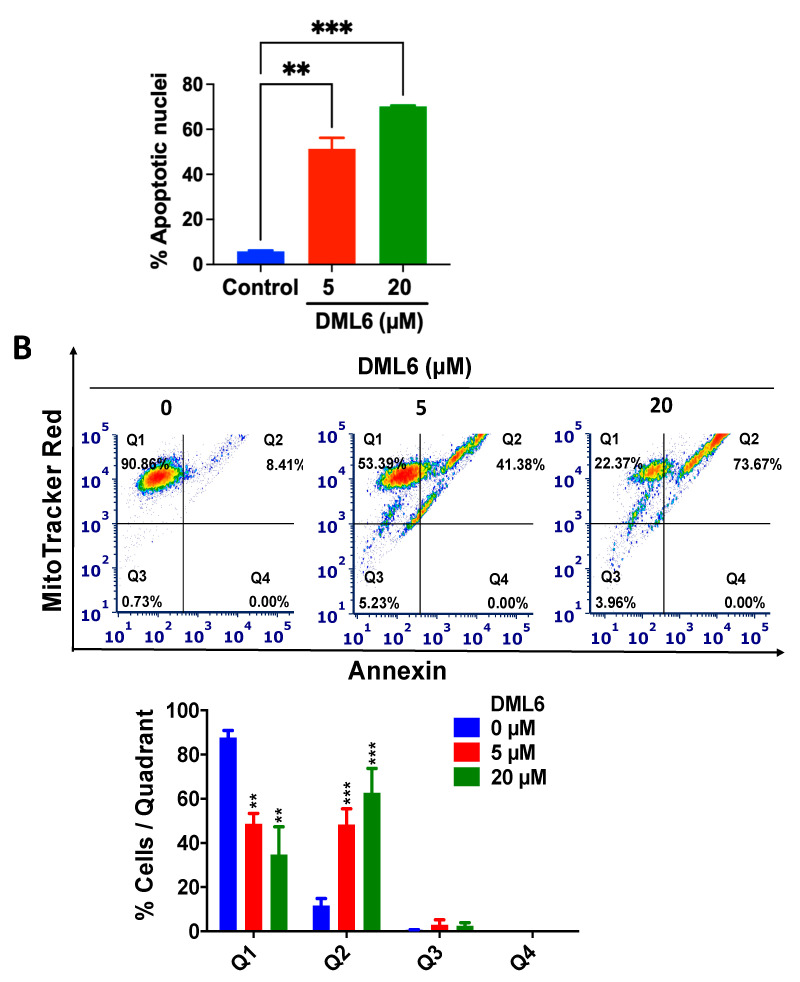
The effect of **DML6** on apoptosis and the mitochondrial membrane potential. (**A**) Changes in the nuclear morphology in OV2008 cells incubated with the vehicle (0 μM), 5, or 20 μM of **DML6**. The cells were fixed and stained with the DNA binding dye, Hoechst 33342. The nuclear fragmentation and apoptotic chromatin condensation are indicated by red arrows. Multiple decondensed micronuclei were observed using an EVOS fluorescent microscope at 20× magnification. Scale bar represents 200 µm. A histogram quantitatively summarizing the % of apoptotic nuclei of cells incubated with 5 and 20 μM of **DML6** as compared to cells incubated with the vehicle, is also shown. (**B**) A representative figure showing **DML6** induced apoptosis A histogram summarizing the results is also shown, ** *p* < 0.01, *** *p* < 0.001. The data represent the means ± SEM of three independent experiments performed in triplicate.

In vitro, **DML6** produced morphological features indicative of apoptosis in the OV 2008 cells, including cell shrinkage, cellular membrane blebbing and the formation of apoptotic bodies, as well as arrest of the cell cycle at the G2 phase. Therefore, we also determined the effect of **DML6** on the nuclear morphology of OV2008 cells, using the Hoechst 33342 dye. The nuclear changes in OV2008 cells after incubation with the vehicle, 5 or 20 μM of **DML6** for 24 were visualized and recorded (Figure 5). As shown in Figure 5, the cells incubated with the vehicle had a normal nuclear shape, consisting of an oval, non-condensed shape, with a low level of bright-blue staining (indicative of viable cells). However, the incubation of cells with 5 μM of **DML6** for 24 h increased the number of cells with condensed, fragmented nuclei, indicating apoptosis. The incubation of OV2008 cells with 20 µM of **DML6** resulted in decondensed, multiple micronuclei and some single, highly condensed nuclei (Figure 5A), indicative of mitotic catastrophe and apoptosis, respectively. The percentage of apoptotic nuclei produced as a result of **DML6** incubation is significantly higher than control incubated with the vehicle. The prominent morphological hallmarks of apoptosis are the presence of apoptotic bodies, i.e., nuclear fragmentation and chromatin condensation [33]. In contrast, mitotic catastrophe (MC) is characterized by the presence of nuclei consisting of two or more lobes or micronuclei in a single cell [34]. Cells incubated with various chemotherapeutic drugs die in the interphase or become arrested at G1 and/or G2 phase [35,36]. The inability of these cells to repair the DNA damage is due to an impairment in checkpoint functions, which causes cells to enter into early mitosis [34]. Their fate is dependent on various conditions, which produces MC, where the cells become viable for a short period of time without replicative capacity (known as permanent growth arrest) or apoptosis and eventually, cell death [34,37,38,39]. Interestingly, apoptosis and MC have similar biochemical features, such as mitochondrial outer membrane permeabilization (MOMP) and the activation of certain caspases [40]. The loss of the MOMP is detrimental as several proteins involved in apoptosis, such as the apoptotic protease-activating factor (APAF-1) and cytochrome c are present in the space between the outer and inner membrane of mitochondria [41]. This results in the loss of mitochondria membrane potential and the disruption of mitochondria function, producing cell death [42,43]. Therefore, the apoptogenic potential of a compound can be determined by its efficacy to induce a loss of the mitochondrial membrane potential. Therefore, we evaluated the induction of apoptosis using MitoTracker™ Red & Annexin V Alexa Fluor^®^ 488 in OV2008 cells. During apoptosis, phosphatidylserine (PS) is translocated to the extracellular side from its regular intracellular mitochondrial localization, leading to phosphatidylserine being present on the extracellular surface [44,45]. The exposed PS binds with high affinity to the fluorophore-labeled human vascular anticoagulant protein, annexin V [46]. The fluorescence intensity is positively correlated with the magnitude of apoptosis induction [47]. As shown in Figure 5B, the majority of OV2008 cells incubated with the vehicle were primarily in quadrant I (90.86%), which contains viable cells and only 8.41% of the cells were present in quadrant II, containing apoptotic cells. After incubation with **DML6**, the percentage of live cells in quadrant I decreased to 53.39% (*p* < 0.01) for 5 µM of **DML6** and 22.37% (*p* < 0.01) for 20 µM of **DML6**. The percentage of apoptotic cells in quadrant II increased to 41.38 % (*p* < 0.001) for 5 µM of **DML6** and 73.67 % (*p* < 0.001) for 20 µM of **DML6** (Figure 5B). The loss of the mitochondrial membrane potential is indicated by a significant shift in the percentage of cells from quadrant I to quadrant II, and this was dependent on the concentration of **DML6**. Overall, the results indicate that **DML6** induces cell death by inducing apoptosis and MC in OV2008 cancer cells.

### 2.7. ***DML6*** Produces a Concentration-Dependent Increase in the Induction of Apoptosis by Activating the Intrinsic Apoptotic Pathway

**Figure 6:**
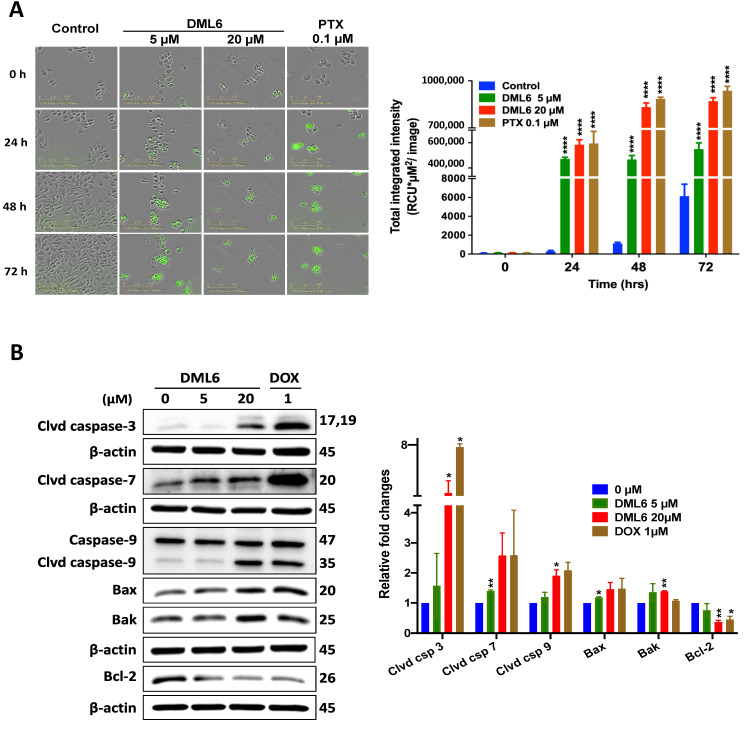
The effect of **DML6** on the induction of intrinsic apoptosis. (**A**) Fluorescence time-dependent experiments to measure the effect of **DML6** on the levels of apoptosis in OV2008 cells. Representative pictures of the fluorescence level of Cell Event™ Caspase-3/7 Green reagent after 24, 48, and 72 h of incubation. Bar = 100 µm. A histogram quantitatively summarizing apoptosis induction at different times for the vehicle, 5 or 20 μM of **DML6**, is also shown. The data were analyzed using a two-way ANOVA analysis of variance, followed by Bonferroni multiple comparison test; **** *p* < 0.0001. (**B**) Western blots for the proteins cleaved caspase-3, cleaved caspase-7, caspase-9, cleaved caspase-9, Bax, Bak and Bcl-2, following overnight incubation with 20 μM of **DML6**. The values of the proteins were normalized to β-actin levels. A histogram summarizing the levels of each protein is also shown. All data are presented as the means ± SEM of four independent studies with * *p* < 0.05, ** *p* < 0.01 vs. control group. Clvd = cleaved; Csp = caspase.

Apoptosis, a form of programmed cell death that results in cell death, is one of the major mechanisms by which chemotherapeutic drugs achieve their therapeutic efficacy [48]. Morphologically, apoptosis is characterized by cytoplasmic and nuclear shrinkage, *chromatin condensation* at the *nuclear* periphery, nuclear fragmentations and blebbing of the plasma membrane [49]. Subsequently, this leads to the production of small apoptotic bodies that have an intact cellular membrane and unaltered integrity of the organelles [50]. These apoptotic bodies are then released and eliminated by phagocytosis in the extracellular environment [51]. Apoptosis can occur in cells by the activation of either the extrinsic or intrinsic pathway. The intrinsic pathway, also known as the mitochondrial pathway, is activated in response to intrinsic stimuli such as DNA damage and cellular stress, and this signal is transmitted to the outer mitochondrial membrane (OMM) by proteins in the Bcl-2 family [52]. The Bcl-2 protein family consists of 3 subclasses of proteins: pro-survival/anti-apoptotic proteins, Bcl-2, Bcl-X_L_, Bcl-W, A1 and Mcl-1, which bind to and sequester another class of proteins, the pore-forming proteins, Bax and Bak and pro-apoptotic BH3-only proteins, Bid, Bim, Bad, Hrk, Bik, Puma and Noxa [53,54]. The balance between the pro-apoptotic and anti-apoptotic proteins determines whether the cell dies or survives [55]. In normal cells, Bak is present in the OMM, whereas the majority of Bax is located in the cytosol and a small fraction is weakly bound to OMM [56]. Upon exposure to intrinsic stimuli, Bax is translocated from the cytosol to OMM, where Bax and Bak are oligomerized to their active conformational form [55]. This leads to the formation of pores in the mitochondria, causing OMM permeabilization and damage. As a result, several apoptogenic molecules, such as cytochrome c and DIABLO/Smac, are released from the mitochondrial membrane space to the cytosol which, in turn, activates the aspartate-specific proteases, known as caspases [51]. First, the initiator caspases, caspase-2, -8, -9, -10, are activated, followed by the subsequent cleavage and activation of the executioner caspases (caspase-3, -6, -7), which ultimately activates several cascades of proteins that produce cell death [57]. To determine whether **DML6**-induced apoptotic death in OV2008 cells occurs by activation of the intrinsic pathway, we performed a real-time quantification of apoptosis using the Caspase-3/7 Green reagent. The incubation of OV2008 cell with 5 or 20 μM of **DML6** significantly increased annexin V green fluorescence over time compared to the OV2008 cells incubated with the vehicle (Figure 6A). A significant difference in fluorescence intensity was observed between the control and **DML6** after 24 hours of incubation, indicating a significant apoptosis induction (Figure 6A). **DML6**, at 5 or 20 μM, significantly induced apoptosis in OV2008 cells in a time-dependent manner by activating caspase-3 and caspase-7, compared to cells incubated with the vehicle (5 and 20 µM, *p* < 0.0001) (Figure 6A). The lowest concentration of **DML6** (5 µM) required a longer incubation time (≈48 h) to induce apoptosis, compared to 20 µM of **DML6** (<24 h; Figure 6A). These results indicate that **DML6** induces apoptotic cell death at early time points. Furthermore, we analyzed the levels of certain intrinsic apoptotic proteins using Western blotting. Our results indicate that 20 μM of **DML6** significantly decreased (*p* < 0.01) the levels of the anti-apoptotic protein, Bcl-2, and 5 and 20 μM of **DML6** significantly increased the expression of the pro-apoptotic proteins, Bax (*p* < 0.05) and Bak (*p* < 0.01). Furthermore, the incubation of OV2008 cells for 24 hours with 20 μM of **DML6** induced the cleavage of initiator caspase-9, compared to cells incubated with the vehicle. The incubation of OV2008 cells with 5 or 20 µM of **DML6** significantly increased the cleavage (and thus, the activation) of caspase-7 (*p* < 0.05) and caspase-3 (*p* < 0.01), respectively, compared to OV2008 cells incubated with the vehicle. These results indicate that **DMl6** produces a significant induction of intrinsic apoptosis in OV2008 cells. Overall, these results indicate that the anticancer efficacy of **DML6** is due, in part, to the induction of intrinsic apoptosis.

Thus, our findings suggest that **DML6** may be a promising lead compound for pre-clinical development of anti-cancer agent against cervical cancer. However, there are some limitations to this study. The preliminary screening of compounds was performed in only two cervical cancer cell lines: OV2008 and HeLa cells. The cervical cancer cell line OV2008 used for this study was previously misidentified as an ovarian cancer model [58]. In 2012, genotypic profiling by Korch et. al revealed that OV2008 was identical to another cervical cancer cell line called ME-180 and was found to be HPV positive [59]. On the other hand, HeLa cells were not chosen for further in vitro experiments due to its history of cross-contamination with other cell lines [60,61]. More mechanistic studies should be conducted on more relevant cervical cancer cell lines. Similarly, in-vivo study using an appropriate cervical cancer model is required to determine whether **DML6** is an efficacious and safe anti-cancer agent in vivo. Additionally, the nature of DNA damage (direct or indirect) induced by **DML6** was not studied in this study, its target identification and effect on DNA damage response signaling pathway along with alterations in cell cycle pathways needs further investigation.

## 3. Materials and Methods

### 3.1. Chemistry

All reagents and solvents used in the synthesis reactions were obtained from commercial sources and used without further purification. The progress of chemical reactions was monitored by thin layer chromatography (TLC), performed on pre-coated aluminum plates of silica gel 60 F254. The developed TLC plates were visualized for compound spots in UV light (254 nm) and/or by exposing the plates to iodine vapor. The separation of the compounds was conducted using column chromatography with silica gel (60–120 mesh). The melting points of the synthesized compounds were measured by an open capillary method on a semi-automatic digital melting point apparatus (Systonic, S-972, Panchkula, India) and the melting points were uncorrected. FTIR spectra were recorded using a spectrophotometer (Perkin Elmer, Spectrum RX-IFTIR). The ^1^H-NMR spectra were recorded in a CDCl_3_ run on a Bruker Avance-II (400 MHz) spectrometer (Bruker, Billerica, MA, USA), and tetramethylsilane (TMS) was used as the internal standard. The chemical shifts were reported in ppm relative to TMS and the coupling constants (*J*) were reported in Hz. The LCMS mass spectra were recorded on a Q-Tof Micro mass spectrometer with an electrospray ionization (ESI) interface (Waters, Milford, MA, USA) in the positive mode. 

#### 3.1.1. General Procedure for the Synthesis of Dialkylamino Substituted Chalcones (**DML1**–**DML6**)

Equimolar mixtures of substituted acetophenone 1a–f (15 mmol) and dialkylamino substituted benzaldehyde 2a–f (15 mmol) in ethanol (25 mL) were stirred for 10 min. Then, 40% NaOH (5 mL) was added dropwise, with continuous stirring for 2–8 h, at room temperature. Subsequently, the reaction mixture was poured into crushed ice (200 g) and neutralized with 10% HCl. The solid precipitate was filtered, washed with cold water and dried in air. The crude product was purified by recrystallization using absolute alcohol.

*(E)-3-[4-(dimethyl amino)phenyl]-1-(pyridin-2-yl)prop-2-en-1-one* (**DML1**). Mp: 126 °C; IR (KBr) ν (cm^−1^): 3100, 1650, 1521, 1432, 1347; 1H NMR (400 MHz, CDCl_3_): δ 8.70 (s, 1H), 8.19 (d, 1H, *J* = 7.5), 8.09 (d, 1H, *J* = 16.0), 7.94 (d, 1H, *J* = 16.0), 7.85 (dd, 1H, *J* = 7.0), 7.65 (dd, 2H, *J* = 8.0), 7.44 (t, 1H, *J* = 19.0), 6.68 (dd, 2H, *J* = 8.5), 3.04 (s, 6H); ESIMS (*m/z*): 253.13 [M + H]^+^.

*(E)-3-[4-(diethyl amino)phenyl]-1-(pyridin-2-yl)prop-2-en-1-one* (**DML2**). Mp: 142 °C; IR (KBr) ν (cm^−1^): 3056, 1654, 1518, 1428, 1352; 1H NMR (400 MHz, CDCl_3_): δ 8.47 (s, 1H), 8.22 (d, 1H, *J* = 7.5), 8.14 (d, 1H, *J* = 16.0), 7.94 (d, 1H, *J* = 16.0), 7.89 (dd, 1H, *J* = 7.0), 7.62 (dd, 2H, *J* = 8.0), 7.38 (t, 1H, *J* = 19.0), 6.61 (dd, 2H, *J* = 8.0), 3.02 (s, 6H); 1.01 (m, 4H); ESIMS (*m/z*): 281.17 [M + H]^+^.

*(E)-3-[4-(dimethyl amino)phenyl]-1-(4-methoxy phenyl)prop-2-en-1-one* (**DML3**). Mp: 118 °C; IR (KBr) ν (cm^−1^): 3081, 1605, 1527, 1433, 1252; 1H NMR (400 MHz, CDCl_3_): δ 8.21 (d, 2H, *J* = 8), 8.14 (d, 1H, *J* = 16.0), 7.74 (d, 1H, *J* = 16.0), 7.59 (d, 2H, *J* = 8.0), 7.32 (d, 1H, *J* = 8.0), 6.98 (d, 1H, *J* = 19.0), 6.60 (d, 2H, *J* = 8.5), 3.90 (s, 3H), 3.01 (s, 6H); ESIMS (*m/z*): 282.16 [M + H]^+^.

*(E)-1-[3,4-dimethoxy phenyl]-3-(4-(dimethyl amino)phenyl)prop-2-en-1-one* (**DML4**). Mp: 86 °C; IR (KBr) ν (cm^−1^): 3052, 1622, 1540, 1429, 1238; 1H NMR (400 MHz, CDCl_3_): δ 8.19 (d, 2H, *J* = 8), 8.02 (d, 1H, *J* = 16.0), 7.54 (d, 1H, *J* = 16.0), 7.23 (d, 2H, *J* = 8.0), 7.02 (d, 1H, *J* = 8.0), 6.80 (d, 1H, *J* = 19.0), 6.67 (d, 2H, *J* = 8.5), 3.91 (s, 3H), 3.78 (s, 3H), 3.06 (s, 6H); ESIMS (*m/z*): 312.24 [M + H]^+^.

*(E)-1-[3,4-dimethoxy phenyl]-3-(4-(dimethyl amino)phenyl)prop-2-en-1-one* (**DML5**). Mp: 110 °C; IR (KBr) ν (cm^−1^): 3004, 1648, 1534, 1428, 1242; 1H NMR (400 MHz, CDCl_3_): δ 8.12 (d, 2H, *J* = 8), 7.89 (d, 1H, *J* = 16.0), 7.63 (d, 1H, *J* = 16.0), 7.23 (d, 2H, *J* = 8.0), 6.91 (d, 1H, *J* = 8.0), 6.87 (d, 1H, *J* = 19.0), 6.67 (d, 2H, *J* = 8.5), 3.87 (s, 3H), 3.07 (s, 4H), 1.12 (s, 6H); ESIMS (*m/z*): 311.2 [M + H]^+^.

*(E)-1-[2,4-dichloro phenyl]-3-(4-(dimethyl amino)phenyl)prop-2-en-1-one* (**DML6**). Mp: 73 °C; IR (KBr) ν (cm^−1^): 3100, 1646, 1568, 1434; 1H NMR (400 MHz, CDCl_3_): δ 8.50 (s, 1H), 8.12 (d, 1H, *J* = 7.5), 8.07 (d, 1H, *J* = 16.0), 7.84 (d, 1H, *J* = 16.0), 7.54 (dd, 1H, *J* = 7.0), 6.85 (dd, 2H, *J* = 8.0), 6.66 (dd, 2H, *J* = 8.5), 3.02 (s, 6H); ESIMS (*m/z*): 321.22 [M + H]^+^.

#### 3.1.2. General Procedure for the Synthesis of Dialkylamino Substituted 1,3,5-Triphenyl-4,5-dihydro-1*H*-pyrazole (**DML7**–**DML12**)

The substituted chalcones **DML1**–**DML6** (5 mmol) and phenylhydrazine (5 mmol) in acetic acid (20 mL) were boiled under reflux for 6 h. After completion of the reaction as indicated by TLC, the reaction mixture was left to cool at room temperature. The solid separated from reaction mixture was filtered, washed with water and dried in air. The crude product was purified by recrystallization or silica-gel column chromatography in a mixture of hexane and ethyl acetate (9:1) as eluent.

*N,N-dimethyl-4-[1-phenyl-5-(pyridin-2-yl)-4,5-dihydro-1H-pyrazol-3-yl]aniline* (**DML7**). Mp: 179 °C: IR (KBr) ν (cm^−1^): 3320, 1587, 1490, 1442; 1H NMR (400 MHz, MHz, CDCl_3_): δ 8.85 (d, 1H, *J* = 1.5), 8.53 (dd, 1H, *J* = 5), 8.28 (m, 1H), 7.54 (m, 1H), 7.16 (m, 4H), 7.06 (dd, 2H, *J* = 7.5), 6.94 (d, 2H, *J* = 8.5), 6.83 (t, 1H, *J* = 3), 5.36 (dd, 1H, *J* = 7), 3.78 (dd, 1H, *J* = 12.5), 3.07 (dd, 1H, *J* = 7), 2.96 (s, 6H); ESIMS (*m/z*): 343.17 [M + H]^+^.

*N,N-diethyl-4-[1-phenyl-5-(pyridin-2-yl)-4,5-dihydro-1H-pyrazol-3-yl]aniline* (**DML8**). Mp: 188 °C: IR (KBr) ν (cm^−1^): 3377, 1579, 1488, 1436; 1H NMR (400 MHz, MHz, CDCl_3_): δ 8.68 (d, 1H, *J* = 1.5), 8.51 (dd, 1H, *J* = 5), 8.24 (m, 1H), 7.60 (m, 1H), 7.16 (m, 4H), 7.12 (dd, 2H, *J* = 7.5), 6.98 (d, 2H, *J* = 8.5), 6.82 (t, 1H, *J* = 3), 5.42 (dd, 1H, *J* = 7), 3.78 (dd, 1H, *J* = 12.5), 3.07 (dd, 1H, *J* = 7), 3.02 (m, 2H,), 1.01 (s, 6H); ESIMS (*m/z*): 371.22 [M + H]^+^.

*4-(5-[4-methoxyphenyl]-1-phenyl-4,5-dihydro-1H-pyrazol-3-yl)-N,N-dimethylaniline* (**DML9**). Mp: 147 °C: IR (KBr) ν (cm^−1^): 3320, 1568, 1483, 1440; 1H NMR (400 MHz, MHz, CDCl_3_): δ 7.65 (d, 2H, *J* = 1.5), 7.23 (m, 4H), 7.06 (m, 2H), 6.91 (dd, 2H, *J* = 7.5), 6.71 (d, 2H, *J* = 8.5), 6.67 (t, 1H, *J* = 3), 5.13 (dd, 1H, *J* = 7), 3.81 (s, 3H), 3.69 (dd, 1H, *J* = 7), 3.10 (s, 1H), 2.90 (s, 6H); ESIMS (*m/z*): 372.12 [M + H]^+^.

*4-(5-(3,4-dimethoxyphenyl)-1-phenyl-4,5-dihydro-1H-pyrazol-3-yl)-N,N-dimethylaniline* (**DML10**). Mp: 134 °C: IR (KBr) ν (cm^−1^): 3316, 15548, 1478, 1442; 1H NMR (400 MHz, MHz, CDCl_3_): δ 7.49 (d, 1H, *J* = 1.5), 7.18 (m, 4H), 7.10 (m, 2H), 7.03 (dd, 1H, *J* = 7.5), 6.83 (dd, 1H, *J* = 7.5 Hz), 6.76 (t, 1H, *J* = 8.5), 6.69 (d, 2H, *J* = 7), 5.17 (dd, 1H, *J* = 7), 3.97 (s, 3H), 3.89 (s, 3H), 3.78 (m, 1H), 3.10 (m, 1H), 2.90 (s, 6H); ESIMS (*m/z*): 402.12 [M + H]^+^.

*N,N-diethyl-4-(5-(4-methoxyphenyl)-1-phenyl-4,5-dihydro-1H-pyrazol-3-yl)aniline* (**DML11**). Mp: 121 °C: IR (KBr) ν (cm^−1^): 3351, 1550, 1447, 1428; 1H NMR (400 MHz, MHz, CDCl_3_): δ 7.66 (d, 2H, *J* = 7), 7.08 (m, 6H), 7.03 (dd, 2H, *J* = 7.5), 6.91 (dd, 2H, *J* = 7.5), 6.75 (t, 1H, *J* = 8.5), 6.61 (d, 2H, *J* = 7), 5.13 (dd, 1H, *J* = 7), 3.81 (s, 3H), 3.76 (m, 1H), 3.32 (m, 4H), 3.07 (m, 1H), 1.14 (s, 6H); ESIMS (*m/z*): 402.26 [M + H]^+^.

*4-(5-(2,4-dichlorophenyl)-1-phenyl-4,5-dihydro-1H-pyrazol-3-yl)-N,N-dimethylaniline* (**DML12**). Mp: 124 °C: IR (KBr) ν (cm^−1^): 3017, 1542, 1469, 1446; 1H NMR (400 MHz, MHz, CDCl_3_): δ 7.80 (d, 1H, *J* = 7), 7.38 (d, 1H, *J* = 7), 7.23 (d, 1H), 7.18 (m, 4H), 7.07 (dd, 2H, *J* = 7.5), 6.80 (t, 1H, *J* = 8.5), 6.68 (d, 2H, *J* = 7), 5.20 (dd, 1H, *J* = 7), 3.76 (m, 1H), 3.98 (m, 1H), 3.31 (m, 1H), 2.91 (s, 6H); ESIMS (*m/z*): 410.01 [M + H]^+^.

### 3.2. Biological Studies

#### 3.2.1. Cell Lines and Cell Culture

A panel of cancer cell lines, including cervical (OV2008 and HeLa), breast (MDA-MB-231), lung (A549) and colon (LOVO), prostate (DU145), human embryonic kidney cells (HEK293), human colon fibroblast cells (CRL1459), and Chinese hamster ovary cells (CHO), were grown as adherent monolayers in flasks containing Dulbecco’s modified Eagle medium (DMEM), supplemented with 10% fetal bovine serum (FBS) and 1% penicillin and streptomycin, in a humidified incubator with 5% CO_2_ at 37 °C.

#### 3.2.2. MTT Assay

The cytotoxic efficacy of the novel derivatives in the cancer cell lines was determined using the 3-(4,5-dimethylthiazol-2-yl)-2,5-diphenyltetrazolium bromide (MTT) assay, as previously described [62,63]. The cells were seeded evenly (180 μL/well) in 96-well plates, at a density of 3000–5000 cells/well and incubated with serial dilutions of the compounds ranging from 0.1 to 100 μM. The MTT dye (4 mg/ml) was added after 72 h of incubation and incubated with the cells for an additional 4 h at 37 °C, allowing the viable cells to biotransform the yellow-colored MTT into dark-blue formazan crystals. Following incubation, the medium was aspirated, and the formazan crystals were dissolved by adding 100 μL of DMSO to each well. Cytation™ 5 and Cytation™ 7 multi-mode detector (Bio Tek Instruments, Winooski, VT, USA) was used to determine the absorbance readings at a wavelength of 570 nm. The IC_50_ values were determined based on 3 separate experiments, with each experiment carried out in triplicate. The selectivity of the compounds was determined by comparing their IC50 values in cervical cancer compared to the normal epithelial cell lines, HEK293, CHO, and CRL1459.

#### 3.2.3. Time-Dependent Cytotoxicity Assays

##### IncuCyte™ Live-Cell Morphology Study

Real-time live cell analysis was performed as previously described [64]. In order to determine the morphological changes induced by **DML6**, OV2008 cells were seeded at 4000 cells/well in a 96-well plate and incubated overnight at 37 °C, with 5% CO_2_ in an incubator. Subsequently, the cells were incubated with 1, 3 or 30 µM of **DML6** or vehicle (DMEM media with 10% FBS and 1% penicillin and streptomycin) and placed in an IncuCyte^®^ S3 Live-Cell Analysis System (Ann Arbor, MI, USA). IncuCyte was then programmed to capture the images at different time points (0, 24, 48 and 72 h), using the integrated IncuCyte S3 software version 2020B (Essen BioScience, Ann Arbor, MI, USA).

##### IncuCyte™ Cytotox Green Assay

The IncuCyte cytotox green reagent (Catalog # 4633, Essence BioScience, Ann Arbour, MI, USA) was used for real time quantification of dead OV2008 cells as previously described [65]. This dye will only penetrate into cells with non-intact membranes (dead or non-viable cells). As the cells die, the increase in cell membrane permeability allows the reagent to enter the nucleus and bind to DNA, which emits green fluorescence at an excitation maximum of 491 nm and emission maximum of 509 nm. OV2008 cells were seeded (100 µL/well) in a 96-well plate and incubated overnight. The following day, **DML6** (0.1–100 μM) was prepared at 3X the final assay concentrations in diluted IncuCyte™ Cytotox Reagent and added to each well (50 µL/well). The cells were immediately placed in the IncuCyte^®^ S3 Live-Cell Analysis System (Essen BioScience, Ann Arbor, MI, USA) and images were taken every 2 h up to 72 h and analyzed using the integrated IncuCyte S3 software version 2020B (Essen BioScience, Ann Arbor, MI, USA).

#### 3.2.4. Time-Dependent Apoptosis Induction Study

Cell Event™ Caspase-3/7 Green Detection Reagent from (Catalog # C10423, Life Technologies, Carlsbad, CA, USA), which is a novel fluorogenic substrate for activated caspase-3/7 that is compatible with living cells, was used to determine apoptosis activation in real-time in OV2008 cells incubated with **DML6**. Briefly, OV2008 cells were seeded in 96-well plates at a density of 1 × 10^3^ cell/well. Twenty-four hours later, the cells were incubated with the vehicle (DMEM media with 10% FBS and 1% penicillin and streptomycin), 5 or 20 µM of **DML6** for 72 h. Five micromolar of the apoptosis-detecting reagents were added to the cells immediately after **DML6** and were incubated for up to 72 h at 37 °C. Fluorescence was determined every 24h using a live cell imaging system (IncuCyte Zoom, Essen Bioscience, Ann Arbor, MI, USA, using an absorption/emission maximum of ~502/530 nm.

#### 3.2.5. Cell Lysis and Western Blot Analysis

The OV2008 cells were lysed to obtain total cellular protein fractions using Mammalian Protein Extraction Reagent (Catalog # 78501, M-PER™, Thermo Fisher Scientific, Waltham, MA, USA). The cells were seeded in 6 mm plates at a density of 1 × 10^6^ and incubated with 5 or 20 µM of **DML6** or 1 µM of doxorubicin (Catalog # T1-2-, TargetMol, Boston, MA, USA) (a positive control) for 24 h. The following day, the cells were washed with ice-cold PBS, scraped using a cell scrapper and collected in Eppendorf tubes. The tubes were centrifuged at 1500 RPM at 4 °C for 5 min. The solution was discarded and replaced with 70 µL of the cell lysis buffer (M-PER reagent, 100 mM Sodium Orthovanadate (NaOV) at a final concentration of 2.5 mM) and a 100X protease inhibitor cocktail (Catalog # P8340, Sigma-Aldrich, St. Louis, MO, USA, final concentration of 1X, containing of aprotinin, bestatin, E-64, leupeptin and pepstatin). Thirty minutes later, the resulting solution was centrifuged at 10,000 RPM at 4 °C for 5 min and the supernatant was collected. The protein concentration of the cell extracts was determined using the bicinchoninic acid (BCA) (Catalog # 786-570, G-Biosciences, Saint Louis, MO, USA) quantification assay. The extracted proteins were loaded and separated onto a 10% tris-glycine gel and the proteins were transferred from the gel onto a 0.45 µM PVDF membrane. After blocking the membranes using 5% milk prepared in Tris-buffered saline containing Tween 20 (TBST) for 1 hour, followed by washing for 15 min, the membranes were incubated overnight with primary antibodies against Rabbit Cleaved caspase-3 (Catalog # 9664S) (1:1000), rabbit Cleaved caspase-7 (Catalog # 8438S)(1:1000), rabbit Caspase 9 (Catalog # 9502S) (1:1000), mouse Cleaved Caspase-9 (Catalog # (1:1000), rabbit Bak (Catalog # 12105T) (1:1000), rabbit Bax (Catalog # 5023T) (1:1000), mouse Bcl-2 (Catalog # 15071S) (1:1000), mouse beta-Actin (Catalog # 3700S) (1:500) (Cell Signaling Technology, Danvers, MA, USA). The following day, the membranes were washed for 30 minutes and incubated for 90 minutes with horseradish peroxidase labeled (HRP) anti-rabbit and anti-mouse secondary antibodies (1:4000 dilutions) in 5% milk prepared in TBST. Subsequently, the membranes were washed for 30 minutes with TBST and developed by SuperSignal™ West Pico PLUS Chemiluminescent Substrate (Thermo Fisher Scientific, Waltham, MA, USA). Subsequently, G:Box Chemi XX6/XX9 (Syngene, Frederick, MD, USA) was used to detect the blots. Finally, the amount of protein in each blot was quantified using the Image J software (NIH, Bethesda, MD, USA). All data were calculated as a ratio to β-actin.

#### 3.2.6. Nuclear Staining Using Hoechst 33258 Dye

Nuclear fragmentation and chromatin condensation were detected using the Hoechst 33258 DNA dye as previously described [66]. OV2008 cells were seeded at a density of 250,000 cells/mL in 6-well plates and incubated overnight at 37 °C. The next day, the cells were incubated with the vehicle (DMEM media with 10% FBS and 1% penicillin and streptomycin), 5 or 20 μM of **DML6** and further incubated overnight at 37 °C. The cells were fixed and stained with the Hoechst 33258 DNA dye for at least 30 min. The stained nucleus fluorescence was detected using an EVOS digital microscope at wavelengths of 460–490 nm.

#### 3.2.7. Cell Cycle Analysis

Cell cycle analysis was conducted using flow cytometry cell cycle analysis with propidium iodide (PI) as previously described [67]. OV2008 cells were plated into 6-well plates at 2.5 × 10^5^ cells/well. The cells were incubated with the vehicle, 5 or 20 μM of **DML6** for 24 h. The next day, the cells were trypsinized with 0.25% trypsin, 2.21 mM EDTA, 1X, washed, counted, and resuspended in 1ml of ice-cold PBS. The cells were then stained with 200 µL (50 µg/ml stock solution propidium iodide (PI) dye and incubated for at least 15 min. The distribution of the cells in each phase of the cell cycle after incubation with the vehicle or **DML6** was determined using A BD FACSCanto™ flow cytometer (BD Biosciences, Becton-Dickinson, San Jose, CA, USA) and analyzed using FCS Express 7 plus De Novo software (Glendale, CA, USA).

#### 3.2.8. Apoptosis and Mitochondrial Membrane Potential Analysis

MitoTracker® Red and Alexa Fluor 488 annexin V kits for flow cytometry (Molecular Probes Inc., Eugene, OR, USA) were used to determine the mitochondrial membrane potential and apoptosis, respectively, in OV2008 cells, as previously described [68]. OV2008 cells were seeded in 6-well plates and incubated with the vehicle, 5 or 20 μM of **DML6** for 24 h. The following day, the cells were trypsinized using 0.25% trypsin, 2.21 mM EDTA, 1×, counted and 4 μL of 10 μM MitoTracker® Red working solution was added to 1 mL of the harvested cells. The cells were incubated at 37 °C with 5% CO_2_ for 30 min. The cells were washed with ice-cold phosphate-buffered saline (PBS), followed by cell resuspension in 100 μL of the annexin binding buffer. The cell suspensions were then incubated with 5 μL of Alexa Fluor 488 annexin V for 15 min, followed by adding 400 μL of the annexin-binding buffer. Finally, flow cytometry was used to detect the fluorescence of stained cells at the following excitation/emission maximum: Alexa Fluor^®^ 488 annexin V: 499/521 nm; MitoTracker® Red: 579/599 nm, using a flow cytometer BD FACSCanto™ (BD Biosciences, Becton-Dickinson, San Jose, CA, USA) and analyzed using FCS Express 7 plus De Novo software (Glendale, CA, USA).

#### 3.2.9. Reactive Oxygen Species (ROS) Detection

The compound, 2′,7′-dichlorofluorescein (H_2_DCFDA), was used to detect ROS as previously described [69]. OV2008 cells were seeded at a density of 250,000 cells/ml. After 24 h of incubation with the vehicle, 5 or 20 µM of **DML6**, the cells were incubated with H_2_DCFDA for 30 min at 37 °C. The cells were washed 3 times with 1X PBS for 5 min. The levels of reactive oxygen species were then determined based on the fluorescence level of the oxidized DCFDA dye (excitation at 485 nm and emission at 535), using a EVOS digital fluorescent microscope at 20× magnification.

#### 3.2.10. Statistical Analysis

The data were statistically analyzed with GraphPad Prism9.1.2 software from GraphPad Software (San Diego, CA, USA). The MTT assay data was analyzed using one-way ANOVA, followed by Bonferroni’s post-hoc analysis. The data from ROS assay and nuclear staining were analyzed using the one-way ANOVA, followed by Dunnett’s post-hoc analysis. Similarly, the statistical analysis of cell cycle assay was computed using two-way ANOVA, followed by Tukey’s post-hoc analysis. The mitochondrial membrane potential and time-dependent apoptosis induction study were performed using two-way ANOVA, followed by Dunnett’s post-hoc analysis, respectively. Finally, the data from the Western blotting experiment were analyzed using unpaired *t*-test. All experiments were repeated in triplicate. The data are expressed as the mean ± the standard error of mean (SEM). The a priori significance level for this study was *p* < 0.05.

## 4. Conclusions

In conclusion, a series of 12 novel chalcone derivatives were designed, synthesized and characterized. After screening these compounds in a panel of cancer cell lines to determine their anti-proliferative efficacy, **DML6**, had the highest in vitro efficacy, with an IC_50_ value of 7.8 µM and had selectivity for inducing cytotoxicity in the cervical carcinoma cell line, OV2008 compared to other epithelial cancer cells i.e., HEK293, LOVO, MDAMB-231, DU-145 and A549. **DML6** induced oxidative stress and arrested the cell cycle at the G2 phase. The incubation of OV2008 cells with **DML6** produced a loss of the mitochondrial membrane potential, resulting in apoptosis and mitotic catastrophe. The apoptotic efficacy of **DML6** was due to its inhibition of the anti-apoptotic protein, Bcl-2, and the upregulation of the pro-apoptotic proteins, Bax and Bak. **DML6** activated caspase-9 and cleaved caspase-3 and-7, producing apoptosis by activating the intrinsic apoptotic pathway. Overall, our results suggest that **DML6** could be a potential lead compound for the pre-clinical development of novel anti-cancer compounds.

## Figures and Tables

**Table 1:** Molecular structures, properties and reaction yields of compounds **DML1**–**DML12**.

Comp Code	Substitution					Molecular Formula	Molecular Weight	Yield %
	X	R_1_	R_2_	R_3_	R_4_			
**DML1**	N	H	H	CH_3_	CH_3_	C_16_H_16_N_2_O	252.32	84
**DML2**	N	H	H	C_2_H_5_	C_2_H_5_	C_18_H_20_N_2_O	280.37	82
**DML3**	CH	H	OCH_3_	CH_3_	CH_3_	C_18_H_19_NO_2_	281.36	86
**DML4**	CH	OCH_3_	OCH_3_	CH_3_	CH_3_	C_19_H_21_NO_3_	311.38	84
**DML5**	CH	H	OCH_3_	C_2_H_5_	C_2_H_5_	C_20_H_23_NO_2_	309.41	85
**DML6**	C-Cl	H	Cl	CH_3_	CH_3_	C_17_H_15_Cl_2_NO	320.21	81
**DML7**	N	H	H	CH_3_	CH_3_	C_22_H_22_N_4_	342.45	79
**DML8**	N	H	H	C_2_H_5_	C_2_H_5_	C_24_H_26_N_4_	370.50	80
**DML9**	CH	H	OCH_3_	CH_3_	CH_3_	C_24_H_25_N_3_O	371.48	74
**DML10**	CH	OCH_3_	OCH_3_	CH_3_	CH_3_	C_25_H_27_N_3_O_2_	401.51	75
**DML11**	CH	H	OCH_3_	C_2_H_5_	C_2_H_5_	C_26_H_29_N_3_O	399.54	78
**DML12**	C-Cl	H	Cl	CH_3_	CH_3_	C_23_H_21_Cl_2_N_3_	410.34	76

**Table 2:** The antiproliferative efficacy of the **DML1**–**DML12** compounds on the proliferation of cancer cell lines (breast, colon, lung, prostate and cervical) and a normal, non-cancerous cell line, HEK293.

Comp Code	IC_50_ ± SD (µM)						
	Kidney	Breast	Colon	Lung	Prostate	Cervical		
	HEK293	MDA-MB- 231	LOVO	A549	DU145	OV2008		
**DML1**	>100	>100	>100	>100	>100	>100
**DML2**	>100	>100	>100	>100	>100	>100
**DML**3	>100	>100	>100	>100	>100	>100
**DML4**	68.5 ± 2.1	26.2 ± 23.9	45 ± 22.6	66.3 ± 4.6	22.5 ± 4.9	13.8 ± 7
**DML5**	>100	>100	94 ± 8.4	>100	>100	79.3 ± 29.2
**DML6**	70 ± 42.4	91.9 ± 11.5	65 ± 49.5	84.9 ± 21.4	96.9 ± 4.4	7.8 ± 0
**DML7**	>100	>100	>100	>100	>100	>100
**DML8**	>100	>100	>100	>100	>100	>100
**DML9**	>100	>100	>100	>100	>100	>100
**DML10**	>100	>100	>100	>100	>100	>100
**DML11**	>100	>100	>100	>100	>100	>100
**DML12**	>100	>100	>100	>100	>100	>100

## Data Availability

The raw data presented in this study is available on request to the corresponding author. Supporting information is provided in the supplementary file.

## Supplementary Materials

The following material is available online, Table S1: The antiproliferative efficacy of the **DML1**–**DML12** compounds on the proliferation of cervical HeLa cancer cell line. Figure S1. The efficacy of **DML6** in HeLa cancer cells.
