# Supplementary material for: A Novel Dialkylamino-Functionalized Chalcone, DML6, Inhibits Cervical Cancer Cell Proliferation, In Vitro, via Induction of Oxidative Stress, Intrinsic Apoptosis and Mitotic Catastrophe
Source: Molecules. 2021 Jul 11;26(14):4214. doi: 10.3390/molecules26144214 (PMC8306139; doi:10.3390/molecules26144214)
Supplement: Supplementary file 1 [file molecules-26-04214-s001.zip › molecules-1234662-supplementary.pdf]

molecules

MDPI

Supplementary Material: A Novel Dialkylamino-Functionalized Chalcone, DML6, Inhibits Cervical Cancer Cell Proliferation, In Vitro, via Induction of Oxidative Stress, Intrinsic Apoptosis and Mitotic Catastrophe

Jenna M Len[1#], Noor Hussein[1#], Saloni Malla[1#], Kyle Mcintosh[1], Rahul Patidar[3], Manivannan Elangovan[3*], Karthikeyan Chandrabose[4], N. S. Hari Narayana Moorthy[4], Manoj Pandey[5], Dayanidhi Raman[2], Piyush Trivedi[6], Amit K. Tiwari [1,2,*]

Table S1. The antiproliferative efficacy of the DML1-DML12 compounds on the proliferation of cervical cancer cell line.

Comp Code	IC_{50} ± SD (μM)
	Cervical HeLa
DML1	70.69 ± 2.19
DML2	>100
DML3	>100
DML4	39.47 ± 11.44
DML5	73.38 ± 4.81
**DML6**	9.08 ± 0.69
DML7	>100
DML8	>100
DML9	>100
DML10	>100
DML11	>100
DML12	>100

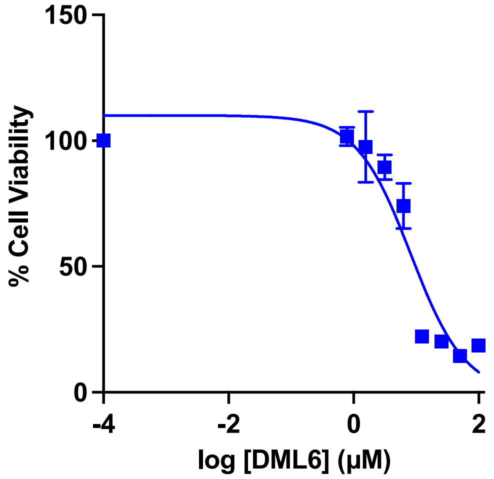

Figure S1. The efficacy of DML6 in HeLa cancer cells. The cell-viability curve produced by HeLa cells treated with vehicle (0 μM), 5 or 10 μM of **DML6** after 72 h of incubation. Cell survival was determined using the MTT assay. The IC_{50} values are represented as the means ± SD of three independent experiments performed in triplicate.
